# Supplementary material for: Carbon-Assistant Nanoporous Gold for Surface-Enhanced Raman Scattering
Source: Nanomaterials (Basel). 2022 Apr 25;12(9):1455. doi: 10.3390/nano12091455 (PMC9102961; doi:10.3390/nano12091455)
Supplement: Supplementary file 1 [file nanomaterials-12-01455-s001.zip › nanomaterials-1642437-supplementary.pdf]

Article

Carbon-Assistant Nanoporous Gold for Surface-Enhanced Raman Scattering

Zhiyu Jing [1], Ling Zhang [1,*], Xiaofei Xu [1], Shengli Zhu [2] and Heping Zeng [3]

[1] School of Optical-Electrical and Computer Engineering, University of Shanghai for Science and Technology, Shanghai 200093, China; 201310032@st.usst.edu.cn (Z.J.); 17625202808@163.com (X.X.)
[2] School of Materials Science and Engineering, Tianjin University, Tianjin 300350, China; slzhu@tju.edu.cn
[3] State Key Laboratory of Precision Spectroscopy, East China Normal University, Shanghai 200241, China; hpzeng@phy.ecnu.edu.cn
* Correspondence: lzhang@usst.edu.cn

.Section characterization of NPG with carbon material for pretreatment after dealloying

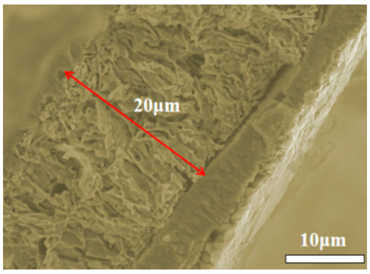

Figure S1.SEM image of the cross section of C@NPG

Morphology characterization and SERS ability comparison of NPG with and without carbon material for pretreatment after dealloying

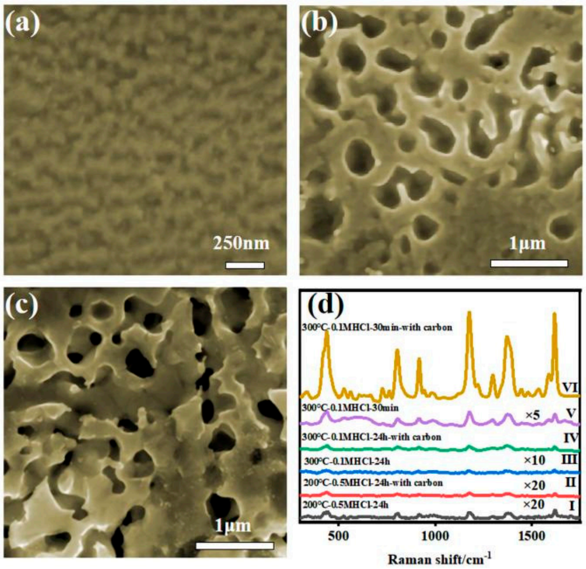

Figure S2. SEM images of NPG prepared with etching precursor alloy (**a**) annealed at 200°C with carbon covering in 0.5M hydrochloric acid for 24 h(**b**) annealed at 300°C without carbon covering and (**c**) annealed at 300°C with carbon covering in 0.1M hydrochloric acid for 24 h; (**d**) Raman spectra of 10^{-6}M crystal violet (CV) molecules adsorbed on NPG prepared at different situations (200 ℃ is the annealing temperature;0.5MHCl is the corrosion

concentration;24h is the corrosion time.) Note: "×5","×10"and "×20" indicate the intensities are magnified by 5 and 20 times for comparison.

Fig S2(a) shows there is no clear porous structure on the surface due to incomplete carbonization of carbon materials, which affects the annealing process of the strip. The porous holes collapse and the ligaments break, which are the result of long-term corrosion in Fig S2(b) and (c).Fig S2(d) was Raman spectra of 10^{-6}M crystal violet (CV) molecules on different substrates. Line Ⅰ is 3.4 times that of line Ⅱ, so carbon materials did not play a gain in the annealing process. By comparing the other lines, the length of corrosion time will affect the integrity of nanopores and ligaments. Moreover, when the corrosion time is 24h, there is still an obvious gain effect with the help of carbon materials(Line IV is 11 times that of line III).In the annealing process, only a small part of the carbon material (weighing paper) turns light yellow at 200 ℃, while the whole turns black at 300 ℃.Therefore, 300 ℃ is more conducive to the carbonization of carbon materials and easier to affect the alloy strip.

Energy dispersive spectrometer analysis

(a)

element	wt%	wt% Sigma
C	2.56	0.77
O	17.07	0.73
S	0.00	0.00
Cu	51.21	1.05
Au	29.16	1.04
total:	100.00	

(b)

element	wt%	wt% Sigma
C	2.13	0.79
O	9.76	0.66
S	0.00	0.35
Cu	38.10	0.94
Au	50.00	1.07
total:	100.00	

Table S1. EDS analysis for surface of (**a**) bare NPG (**b**)carbon-coated NPG

Simulated EM field distribution of C@NPG with carbon layer of different thickness

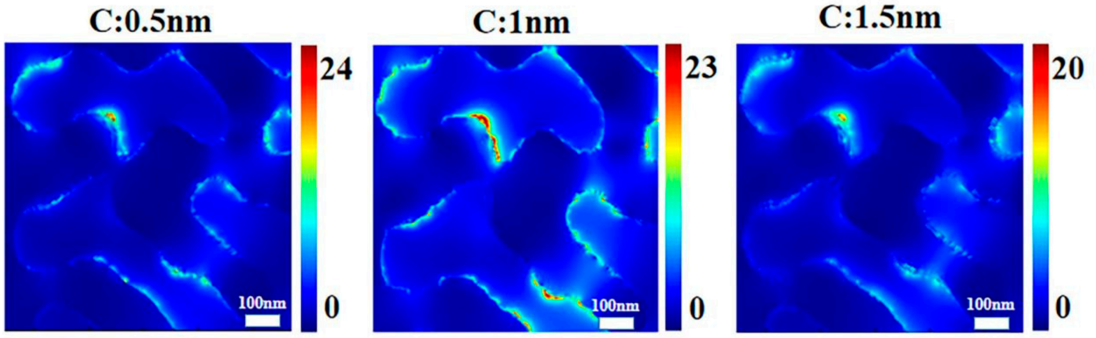

C: the thickness of carbon layer on the ligament surface

Figure S3. EM field intensity variation with the carbon layer thickness of C@NPG.

The porous size of NPG were determined from the SEM images. An ideal model of the NPG was created by randomly etching porous structure.The dielectric constant of Au is obtained through investigation[37],and the background refractive index is 1.The periodic boundary condition was applied to x- and y-directions and the perfectly matched layer (PML) was set in the Z-boundaries.The mesh size was 0.6nm（x）, 0.6nm（y） and 1nm（z） in three

directions around the NPG boundary to obtain the local enhancement of the EM field with high spatial resolution. A power monitor was placed at the x-y plane slightly on the NPG interface to collect the distribution and intensity of the EM field.The excitation wavelength is 633 nm. The thickness of the carbon layer will affect the distance between the molecule and the NPG surface.Therefore, the thickness of the carbon layer is set to 0.5nm, 1nm and 1.5nm for comparison.As shown in Fig. S3, the intensity of EM field decreases with the increase of carbon layer.This change of the SERS EF is likely attributed to strong plasmonic coupling at the nanoligaments between the carbon layer and NPG. Thus, the main factors, which are responsible for the tunable intensity of the EM field, are the thickness of the carbon layer in Fig. S3. Through comparison, we can see that although the strongest EM field intensity slightly decreases with the increasing of the carbon layer thickness, but the one with the carbon layer of 1nm exhibits larger area with stronger EM field.

SERS detection of the 10^{-6}M CV molecules on repreparative C@NPG substrate

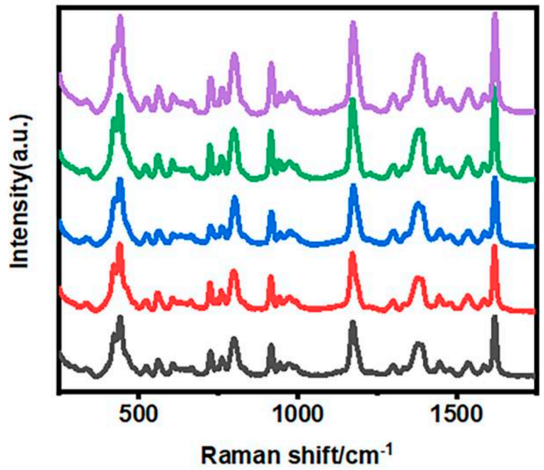

Figure S4. SERS spectra of CV (10^{-6}M) obtained on five different C@NPG substrates

The deviation of signal intensity of C@NPG substrate.

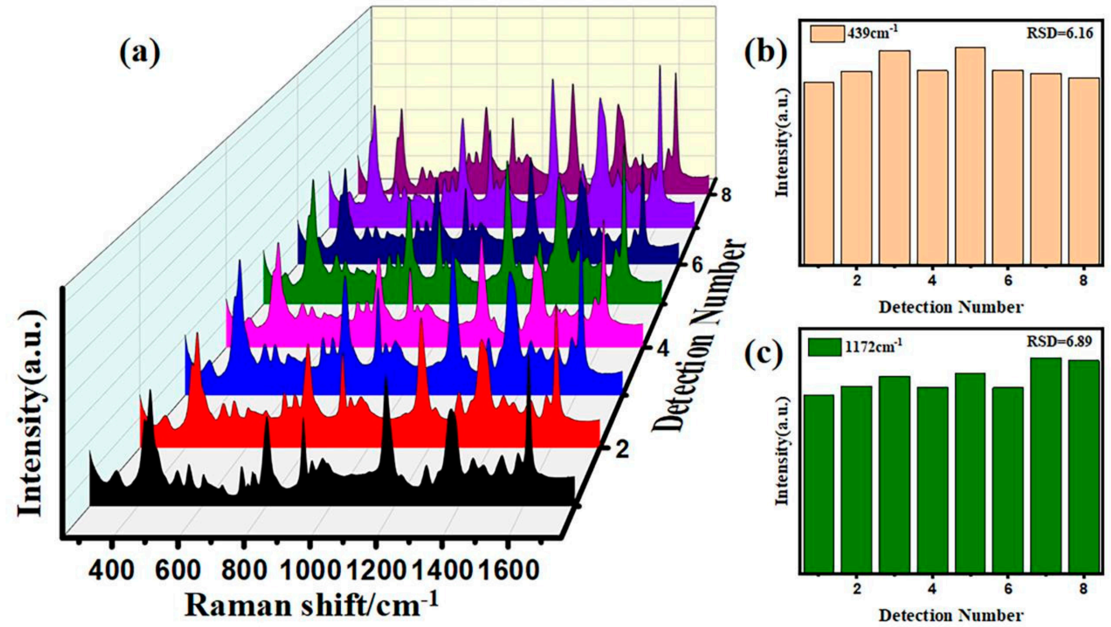

Figure S5 .(**a**) Raman spectra of the 10^{-6}M CV collected from 8 different sites on C@NPG substrate, and (**b**,**c**) the corresponding SERS intensity of characteristic peaks 439 cm^{-1} and 1172 cm^{-1} obtained at the 8 sites. (RSD: relative standard deviation)

Raman spectra of the CV molecules with different concentrations for the C@NPG

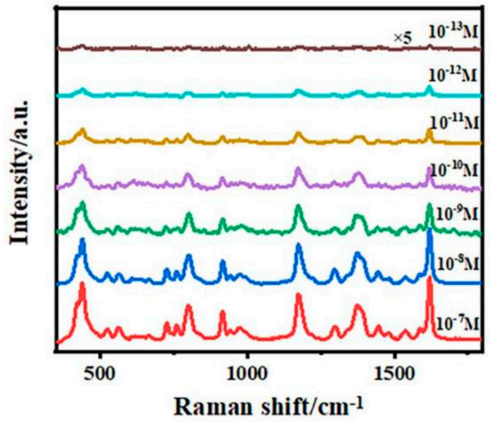

Figure S6. Raman spectra of the CV molecules with different concentrations ranged from 10^{-13} M to 10^{-7} M.

Nicotinamide detection with C@NPG substrate

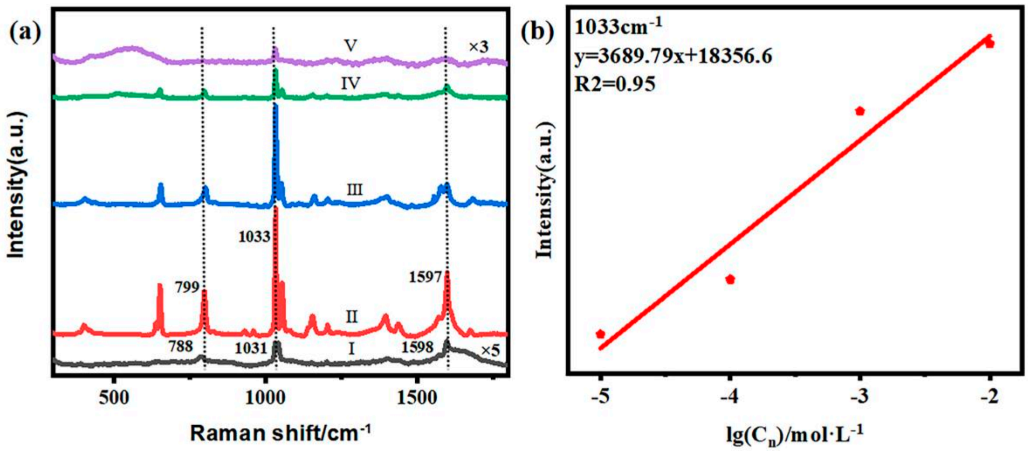

Figure S7. (**a**) Ordinary Raman spectra of nicotinamide (Ⅰ:10^{-2}M aqueous solution) and SERS spectra of nicotinamide with different concentrations (Ⅱ~Ⅴ:10^{-2}M~10^{-5}M); (**b**) SERS intensity as a function of the concentration of nicotinamide : 1033cm^{-1}

A drop of nicotinamide aqueous solution (20μl) was added onto the substrates for SERS detection. A 532 nm excitation laser with a low power of 1.0 mW was adopted for SERS spectra measurement, and integral accumulation time of each Raman spectrum was 10s (temperature 25°C). Line Ⅰ [Fig.S7(a)] is ordinary raman spectra of nicotinamide for 10^{-2}M.788cm^{-1} is O=C-NH_2 vibration, 1031cm^{-1} and 1598cm^{-1} are ring respiration[38]. Three characteristic peaks are measured from aqueous solution. Under the action of substrates, more characteristic peaks can be obtained [from line Ⅱ~Ⅳ in Fig.S7(a)]. Although the Raman characteristic peak has shifted,which belongs to the normal range due to lower than 20cm^{-1}.With the decrease of nicotinamide concentration, the intensity of the characteristic peak at 1033 cm^{-1} gradually decreases, and the corresponding intensity curve is shown in Fig S7(b). The fitting equation is: y =3689.79x + 18356.6, and the correlation coefficient :R^2= 0.95.

Comparison the SERS performance of plasmonic nano gold-based SERS substrate

Table S2. Comparison between this work and previously reported studies for the SERS performance of plasmonic nano gold-based SERS substrate

SERS material	Probe molecule	LOD	EF	Reference
Nanoporous Au thin films	R6G	10^{-8} M	-	[39]
Hierarchical nanoporous gold	CV	10^{-12}M	2.16×10^7	[15]
Homogeneous Au nanorampart arrays	R6G	10^{-8}M	-	[40]
Rough gold nanoarrays	R6G	10^{-12}M	1.9×10^7	[41]
Gradient nanoporous gold	CV and R6G	-	Over 10^7	[13]
Sharp gold nanocones	R6G	10^{-12}M	10^7	[42]
Nanoporous Au films	RhB	10^{-7}M	2.4×10^5	[12]
Carbon-assistant nanoporous gold	CV	10^{-13}M	3.72×10^8	This work

LOD = Limit of Detection

EF = Enhancement Factor
